# Heterogeneity of respiratory dendritic cell subsets and lymphocyte populations in inbred mouse strains

**DOI:** 10.1186/1465-9921-13-94

**Published:** 2012-10-15

**Authors:** Holger Hackstein, Andreas Wachtendorf, Sabine Kranz, Jürgen Lohmeyer, Gregor Bein, Nelli Baal

**Affiliations:** 1 Institute for Clinical Immunology and Transfusion Medicine, Justus-Liebig-University Giessen, Member of the German Center for Lung Research, Langhansstr 7, D-35390, Giessen, Germany; 2 Department of Internal Medicine II, University of Giessen Lung Center, Klinikstrasse 36, D-35392, Giessen, Germany

## Abstract

**Background:**

Inbred mouse strains are used in different models of respiratory diseases but the variation of critical respiratory leukocyte subpopulations across different strains is unknown.

**Methods:**

By using multiparameter flow cytometry we have quantitated respiratory leukocyte subsets including dendritic cells subpopulations, macrophages, classical T and B cells, natural killer cells, γδTCR+ T cells and lineage-negative leukocytes in the five most common inbred mouse strains BALB/c, C57BL/6, DBA/2, 129SV and C3H. To minimize confounding environmental factors, age-matched animals were received from the same provider and were housed under identical specific-pathogen-free conditions.

**Results:**

Results revealed significant strain differences with respect to respiratory neutrophils (p=0.005; up to 1.4 fold differences versus C57BL/6 mice), eosinophils (p=0.029; up to 2.7 fold), certain dendritic cell subsets (p≤0.0003; up to 3.4 fold), T (p<0.001; up to 1.6 fold) and B lymphocyte subsets (p=0.005; up to 0.4 fold), γδ T lymphocytes (p=0.003; up to 1.6 fold), natural killer cells (p<0.0001; up to 0.6 fold) and lineage-negative innate leukocytes (p≤0.007; up to 3.6 fold). In contrast, total respiratory leukocytes, macrophages, total dendritic cells and bronchoalveolar lavage leukocytes did not differ significantly. Stimulation of respiratory leukocytes via Toll-like receptor 4 and 9 as well as CD3/CD28 revealed significant strain differences of TNF-α and IL-10 production.

**Conclusion:**

Our study demonstrates significant strain heterogeneity of respiratory leukocyte subsets that may impact respiratory immunity in different disease models. Additionally, the results may help identification of optimal strains for purification of rare respiratory leukocyte subsets for ex vivo analyses.

## Introduction

Pulmonary host defense is mediated by different types of immunocompetent leukocytes including classical T and B lymphocytes, innate lymphocytes (NK cells, γδ T cells), professional antigen presenting cells (macrophages, dendritic cells) and granulocytes. Recent evidence indicates that these cells are not only stimulators of immunity and inflammation but additionally regulate immune responses and exhibit anti-inflammatory activity. Many respiratory immune responses result in tolerance to subsequent antigen challenge, which is mediated by Foxp3+ regulatory T (Treg) cells interacting with professional antigen presenting cells [1].

Dendritic cells (DC) are rare professional antigen presenting cells playing critical roles as initiators and regulators of innate and adaptive immunity [2-4]. In the murine respiratory tract, distinct DC subsets have been identified [5,6]. These respiratory DC subsets form an interdigitating network of cells being specialized for different immunological functions in the respiratory tract. Respiratory DC can be separated based on the expression of different surface markers in at least four major subsets: plasmacytoid DC (pDC), CD103^+^ DC, CD103^neg^ CD11b^high^ MHC-class-II^high^ DC (CD11b^high^ DC) and CD103^neg^ CD11b^+^ MHC-class-II^neg-med^ monocytic DC (MoDC) [5,7]. Respiratory pDC are not only involved in limitation of viral respiratory infection [8] but additionally prevent airway hyperresponsiveness both after infection [9] and after inhalation of harmless antigens [10]. CD103^+^ DC have been described to express high levels of the Langerhans cell marker langerin and were increased in mice with airway hyperresponsiveness and eosinophilia suggesting a role in allergen-induced respiratory inflammation [11] With respect to adaptive T cell immunity against different pathogens, CD103^+^DC, CD11b^high^ DC and MoDC have been identified as major migratory subsets presenting antigens in the draining lymph nodes to naïve CD4^+^ and CD8^+^ T cells [5,12-15].

Although respiratory DC are central for regulation of lung immunity [16] they closely interact with respiratory T and B cells, innate lymphocytes, such as natural killer (NK) cells and γδ TCR^+^ T (γδ T) cells and myeloid-derived leukocytes, such as respiratory macrophages and granulocytes.

So far, most of the studies have been focused on selected subsets of respiratory leukocytes and a complete analysis including all mentioned subsets is still lacking. Moreover, it is not clear whether different inbred mouse strains with a different genetic background exhibit differences in the respiratory frequencies of these functionally relevant leukocyte subsets. Given the fact, that inbred mouse strains are known for their marked variation of susceptibility and resistance against various pathogens we hypothesized that inbred mouse strains are likely to exhibit major differences of respiratory leukocyte subset frequencies. In this study we have enumerated the major respiratory leukocyte subsets including DC subpopulations, innate and adaptive lymphocytes, respiratory macrophages and granulocytes in the five most common inbred mouse strains C57BL/6, BALB/c, C3H, DBA/2 and 129SV. Additionally, since increasing evidence suggest an important role of innate lineage-negative (lin^neg^) leukocytes in immunity [17,18]we also quantitated the frequencies of respiratory lin^neg^ leukocytes. The results indicate marked differences in the frequencies of respiratory DC subsets, innate and classical lymphocytes and innate lin^neg^ leukocytes providing additional insight into the heterogeneity of inbred mouse strains.

## Material and methods

### Mice

Specific-pathogen-free mice, BALB/c (Balb/cAnNCrl), C57BL/6 (C57BL/6NCrl), DBA/2 (DBA/2NCrl), 129SV (129S2/SVPasCrl) and C3H (C3H/HeNCrl), 7–11 weeks of age were purchased from Charles River, Sulzfeld, Germany and maintained under specific-pathogen-free conditions. The C3H mice do not carry the TLR4^LPS-d^ mutation and are therefore LPS sensitive. Analyses were performed after approval of the regional authority board (animal ethics Giessen A2/2011).

### Lung preparation

Lung single cell suspension were prepared after enzymatic digestion as described in detail elsewhere [5] with minor modifications. Briefly, mice were euthanized and lungs were perfused via the right ventricle with HBSS (PAA, Germany) to remove the intravascular pool of cells. Tissues were minced and digestion was performed in 0.09 U/ml type A collagenase (Roche, Germany) and 9.09 U/ml DNase (Roche, Germany) in IMDM (PAA, Germany) with 10% FCS (PAA, Germany) at 37°C for 1h. Single cell suspension were prepared by tissue resuspension with 20 G 1 ½ canules (0.9 x 40 mm; BD, Germany) and by mashing through a 70 μM cell strainer (BD, Germany). Red blood cells were lysed by ammoniumchloride lysis. Cells were washed with HBSS for flow cytometry staining or leukocytes were magnetic-bead sorted after washing with PBS/2% BSA/2mM EDTA (PAA, Germany). Bronchoalveolar lavages (BAL) were performed as described elsewhere [19]. In order to assess whether lung preparations of inbred mouse strains contained different total CD45+ leukocyte numbers we used the trucount method (BD Biosciences, Germany) to enumerate absolute leucocyte counts per lung preparation. The tubes contain a known number of fluorescent beads allowing the flow cytometer software to calculate absolute cell counts.

### Flow cytometry and staining procedure

Cellular phenotyping was performed on a FACS CantoII flow cytometer (Becton Dickinson, San Jose, CA, USA). The following fluorochrome-labelled monoclonal antibodies conjugated to FITC, PE, PeCy7, PerCPCy5.5, APC, APC-Cy7, Pacific Blue and appropriate isotype controls were used for surface staining according to the manufacturer’s instructions: CD3e, CD4, CD8a, CD11b, CD11c, CD19, CD25, CD45, CD49b, CD90, CD103, CD127, TCR-γδ, I-A/I-E-, GR1, F4/80 (all mabs from Biolegend, Germany), CD39, Foxp3 (eBioscience, Germany), Siglec-F (BD Biosciences, Germany) and 120g8 (Dendritics, France). The lineage cocktail consisted of the following mAbs: CD3e (BD Pharmingen, Germany), CD4, CD8a, CD11b, CD11c, CD19, B220, TER-119, FceR1 (all from Biolegend, Germany). Autofluorescent respiratory macrophages were identified through Siglec-F expression.

A detailed list of mabs panels and fluorochrome combinations is summarized in Table 1 (Table 1). Isotype matched control antibodies were ordered from the same company. Isotype controls were used to control specificity of staining of lineage markers and as fluorescence minus one (FMO) controls to control for specificity of complex subset markers or during intracellular stainings. Surface mAb or isotype staining time was 30 min on ice and cells were washed with staining buffer (1x PBS/5% FBS; both reagents from PAA, Germany) at 400g, 5 min, room temperature (RT) before analysis. For intracellular staining, samples were first stained for surface antigens, washed with permeabilization buffer (ebioscience, Germany) 5 min, RT, 400 g and supernatant was discarded. Cell pellet was vortexed for dissociation and incubated with fixation/permeabilization buffer (anti mouse/rat Foxp3 staining set, ebioscience, Germany) 30 min on ice. 2 ml of permeabilization buffer (eBioscience) was added directly to the pellet before centrifugation (400 g, 5 min, RT). Washing with permeabilization buffer was repeated once. Cells were resuspended in 100 μl permeabilization buffer and intracellular mabs or isotype controls were added at the recommended concentrations and incubated 30 min on ice. Cells were washed two times with permabilization buffer and immediately analysed by flow cytometry. The number of acquired events was ≥ 500,000 after surface or intracellular stainings.

**Table 1:** Antibody panels with fluorochromes and mAbclones

**Panel**		**FITC**	**PE**		**PE-Cy7**	**PerCP-Cy5.5**	**APC**	**APC-Cy7**	**P-Blue**		
**Lympho1**	antigen	TCR gd	CD3e		CD4	CD19	CD8a	CD45	CD49b		
	clone	GL3	145-2C11		GK 1.5	1D3	53-6.7	30-F11	DX5		
	Cat.No.	118106	100307		100406	551001	100712	103116	108918		
	company	Bio-legend	Biolegend		Biolegend	BDPharm.	Biolegend	Biolegend	Biolegend		
**Lympho2/ Lin-neg cells**	antigen	Lin-cocktail	CD3e		CD90.2	CD4		CD45	CD25		
	clone	see	145-2C11		53-2.1	GK 1.5		30-F11	eBio3C7		
	Cat.No.	below	100307		140310	100434		103116	48-0253-82		
	company		Biolegend		Biolegend	Biolegend		Biolegend	eBio-science		
**DC**	antigen	I-A/I-E	SiglecF	CD49b	CD11c	CD103	pDC/IPC-A647	CD45	CD11b		
	clone	M5/114.15.2	E50-2440	DX5	N418	2 E 7	120g8	30-F11	M1/70		
	Cat.No.	107606	562068	108908	117306	121416	DDX039A647	103116	101224		
	company	Bio-legend	Biolegend		Biolegend	Biolegend	Dendritics	Biolegend	Biolegend		
**Mono/**	antigen	I-A/I-E	SiglecF		CD11c	CD11b	GR1	CD45	F4/80		
**Granulo**	clone	M5/114.15.2	E50-2440		N418	M1/70	RB6-8C5	30-F11	BM8		
	Cat.No.	107606	562068		117306	550993	108412	103116	123124		
	company	Bio-legend	Biolegend		Biolegend	BD Pharm.	Biolegend	Biolegend	Biolegend		
**Tregs**	antigen	CD127	CD39		CD3e	CD4	Foxp3	CD45	CD25		
	clone	A7R34	24DMS1		145-2c11	GK1.5	FJK-16s	30-F11	eBio3C7		
	Cat.No.	135009	12-0391-82		100320	100434	77-5775-40	103116	48-0253-82		
	company	Bio-legend	eBioscience		Biolegend	Biolegend	eBioscience	Biolegend	eBio-science		
**LIneage cocktail**		**all FITC**									
	antigen	CD3e	CD4	CD8a	CD45R/B220	TER-119	CD11b	CD11c	CD49b	FceR1	CD19
	clone	145-2C11	GK 1.5	53-6.7	RA3-6B2	TER-119	M1/70	N418	DX5	MAR-1	6D5
	Cat.No.	553058	100406	100706	103206	116206	101206	117306	108906	134306	115506
	company	BD Pharm	Biolegend								

### Cell culture experiments, reagents and cytokine quantification

Lung leukocytes were magnet-bead purified using CD45 microbeads (Miltenyi Biotec, Germany) according to the manufacturer instruction (CD45 purity > 85%). Viability of cells was > 90% as indicated by trypan blue staining. 2x10^5^ respiratory leukocytes from different mouse strains were stimulated in 96-well plates (Greiner, Germany) with TLR4 agonist LPS (1 μg/ml; E coli serotype 0111:B4 strain, Sigma Aldrich, Germany), TLR9 agonist CpG 1826 (3.1 μM/ml; Invivogen, France) for 24h and CD3/CD28 mAb (1 μg/ml each, Biolegend, Germany) for 48h in RPMI 1640 medium supplemented with L-glutamine, penicillin/streptomycin, 10% heat-inactivated FCS (PAA Germany). Mouse TNF-α, IFN-γ and IL-10 were quantitated by ELISA (ElisaMax Standard Set, Biolegend, Germany).

### Statistical analyses

The significance of differences between groups were analysed by one-way ANOVA and Tukey post-test for multiple comparisons. A p-value < 0.05 was considered significant. Data are shown as means (± SEM). Statistical analyses were performed with Prism 5.02 software (Graphpad software, Inc.).

## Results

### Inbred mouse strains exhibit similar numbers of total respiratory leukocytes, macrophages, DC, BAL but different granulocyte numbers

**Figure 1:**
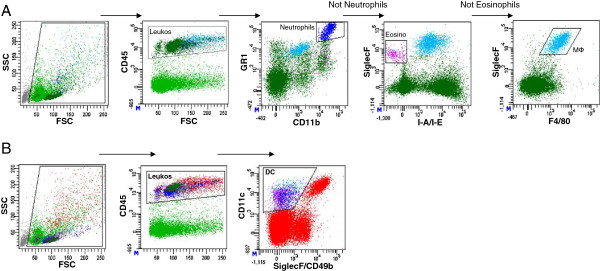
**Gating strategy for identification of respiratory leukocytes, neutrophils, eosinophils and dendritic cells.** Cells were gated based on scatter light (FSC, SSC) characteristics and respiratory leukocytes were identified by CD45 expression (**A**, **B**). Out of the CD45+ cells, neutrophils were identified by Gr1^bright^CD11b^bright^ expression. Subsequently, out of the neutrophil negative fraction, eosinophils were identified by SiglecF^+^MHC-class-II^neg^ (I-A/I-E^neg^) expression. Out of the neutrophil and eosinophil negative fraction macrophages were identified as SiglecF^++^F4/80^+^ double positive cells (**A**). Total dendritic cells were identified as CD11c^+^ SiglecF^neg^ CD49b^neg^ respiratory leukocytes (**B**).

**Figure 2:**
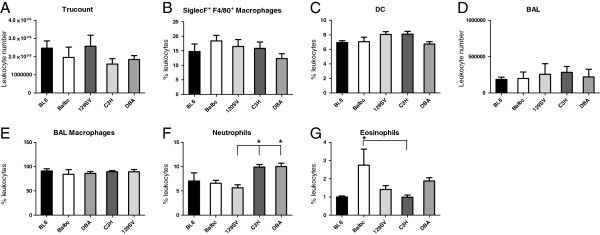
**Inbred mouse strains exhibit similar numbers of total respiratory leukocytes, macrophages, DC, BAL but different granulocyte numbers.** Flow cytometry analysis of lung homogenate and BAL indicates no significant strain differences of total respiratory leukocytes (**A**), macrophage (**B**), and dendritic cell (DC; **C**) frequencies as well as BAL leukocyte numbers (**D**) and BAL macrophage frequencies (**E**). Neutrophil (**F**) and eosinophil frequencies (**G**) exhibit significant strain heterogeneity as indicated. Results are representative for n≥ 8 (**A**, **B**, **C**, **F**, **G**) or n ≥ 5 (**D**, **E**) independent animals per strain (mean ± SEM; *p<0.05; **p<0.01; ***p<0.001).

Respiratory leukocyte subsets were dissected in lung homogenate by multiparameter flow cytometry (Figure 1). After identification of leukocytes through CD45+, respiratory neutrophils were subsequently identified as GR1^high^CD11b^high^, eosinophils as SiglecF^+^ MHC-class-II^neg^ and macrophages as SiglecF^high^ F4/80^+^ cells (Figure 1A). Total respiratory DC were identified as CD11c^+^ SiglecF^neg^CD49b^neg^ leukocytes (Figure 1B). Results indicated that total respiratory leukocyte numbers do not differ significantly between common inbred mouse strains (Figure 2A). Accordingly, in subsequent analyses subset frequencies were quantitated in % of leukocytes. Analysis of respiratory macrophages (Figure 2B), dendritic cells (Figure 2C) and BAL numbers including BAL macrophages (Figure 2D, E) indicated similar results. In contrast, respiratory neutrophil (Figure 2F) numbers were elevated in C3H and DBA mice and eosinophil (Figure 2G) numbers were elevated in BALB/c mice. Magnitude of fold-differences for all leukocyte subsets in comparison to C57BL/6 mice is summarized in Table 2.

**Table 2:** **Fold differences of respiratory leukocyte subset frequencies in comparison to C57BL/6 mice**^**1**^

**Subset**	**BALB/c (± SEM)**	**DBA/2 (± SEM)**	**C3H (± SEM)**	**129SV (± SEM)**
CD45+ leukocytes	0,792 (0,22)	0,746 (0,08)	1,043 (0,24)	0,643 (0,12)
Siglec F^+^ F4/80^+^ macrophages	1,249 (0,12)	0,837 (0,11)	1,075 (0,14)	1,117 (0,16)
Neutrophils	0,935 (0,08)	1,428 (0,09)	1,406 (0,08)	0,799 (0,09)
Eosinophils	2,742 (0,88)	1,882 (0,17)	0,980 (0,12)	1,407 (0,21)
**Dendritic cell (DC) subsets**				
DC	1,015 (0,08)	0,968 (0,04)	1,165 (0,05)	1,159 (0,05)
Plasmacytoid DC	0,954 (0,04)	0,948 (0,06)	0,889 (0,13)	3,423 (0,42)
CD103^+^ DC	1,206 (0,11)	0,723 (0,08)	1,643 (0,15)	0,669 (0,08)
Monocytic DC	0,935 (0,10)	0,962 (0,05)	1,551 (0,11)	1,134 (0,08)
CD11b^hi^ DC	0,914 (0,10)	0,824 (0,13)	0,798 (0,02)	1,543 (0,16)
**Classical lymphocytes**				
CD3^+^ T cells	1,277 (0,03)	0,967 (0,04)	0,830 (0,04)	1,154 (0,09)
CD3^+^ CD4^+^ T helper cells	1,649 (0,07)	1,093 (0,07)	0,959 (0,05)	1,399 (0,12)
CD3^+^ CD8^+^ T cells	0,894 (0,03)	0,691 (0,03)	0,683 (0,03)	0,786 (0,06)
CD4^+^ CD25^+^ T cells	1,489 (0,04)	1,291 (0,10)	1,085 (0,11)	1,626 (0,18)
CD19^+^ B cells	0,557 (0,12)	0,715 (0,06)	0,446 (0,02)	0,504 (0,05)
**Innate lymphocytes and Lin**^**neg**^**leukocytes**				
CD49b^+^ NK cells	0,859 (0,07)	1,127 (0,06)	1,270 (0,03)	0,633 (0,03)
TCR γδ^+^ T cells	1,164 (0,05)	1,636 (0,08)	1,557 (0,18)	1,450 (0,09)
Lin^neg^ CD45^+^ CD90^+^ innate cells	1,480 (0,08)	0,540 (0,05)	0,977 (0,07)	1,194 (0,26)
Lin^neg^ CD45^+^ CD90^neg^ innate cells	2,034 (0,14)	2,185 (0,50)	3,673 (0,66)	1,573 (0,46)
**T regulator cell populations**				
CD4^+^ CD25^high^ T cells	1,179 (0,05)	0,545 (0,02)	0,596 (0,02)	1,014 (0,04)
CD4^+^ CD25^high^ Foxp3^+^ Treg cells	1,089 (0,05)	0,536 (0,02)	0,602 (0,02)	0,986 (0,04)
CD4^+^ CD25^high^ CD39^+^Foxp3^+^ Treg cells	2,000 (0,11)	0,959 (0,05)	1,250 (0,07)	1,656 (0,10)
CD4^+^ CD25^+^ CD127^lo/neg^ Treg cells	1,263 (0,06)	0,535 (0,04)	0,660 (0,03)	1,154 (0,07)
**BAL cells**				
BAL leukocytes	1,071 (0,47)	1,189 (0,55)	1,525 (0,43)	1,381 (0,78)
BAL macrophages	0,926 (0,10)	0,945 (0,04)	0,983 (0,02)	0,981 (0,05)

^1^Results are representative for n≥5 (Lin^neg^ subsets; BAL cells, Treg subsets) or n≥8 (all other subsets) independent animals per strain.

### Significant differences of respiratory dendritic cell subsets across inbred mouse strains

**Figure 3:**
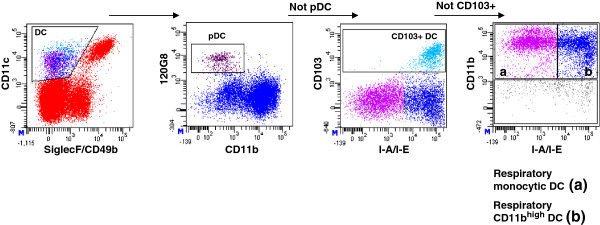
**Gating strategy for identification respiratory DC subsets.** Respiratory dendritic cells (DC) were identified according to CD11c^+^Siglec-F^neg^ CD49b^neg^ expression to exclude autofluorescent macrophages and NK cells and further dissected into plasmacytoid DC (pDC) according to 120g8 expression. The pDC negative DC fraction was gated for CD103 to identify CD103^+^ DC. Out of the pDC and CD103+ DC negative fraction, MoDC and CD11b^high^ DC were separated according to MHC-class-II (I-A/I-E) and CD11b expression. MoDC are CD11b^high^ and MHC-class-II^med-low^ whereas CD11b^high^ DC are CD11b^high^ and MHC-class-II^high^.

**Figure 4:**
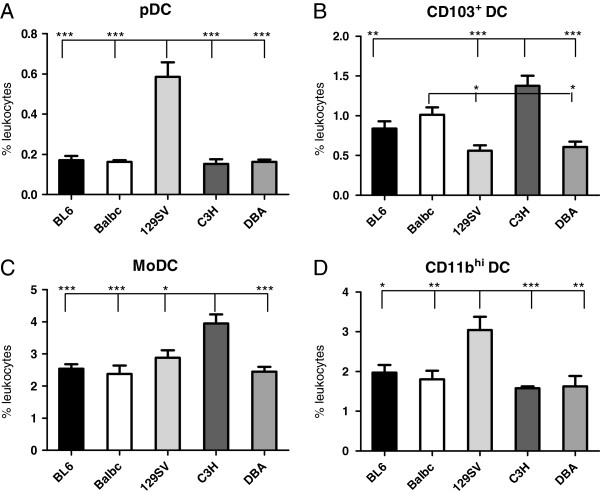
**Marked strain heterogeneity of respiratory DC subsets.** Dissection of respiratory DC subsets with flow cytometry indicates significantly elevated plasmacytoid DC (**A**) numbers in 129SV mice. Respiratory CD103+ DC numbers are significantly elevated in C3H mice when compared to C57BL/6 (BL6), 129SV and DBA mice (**B**). Monocytic DC (MoDC) numbers are significantly elevated in C3H mice when compared to any other strain (**C**). CD11b^high^ DC numbers are significantly elevated in 129SV mice compared to any other strain. Results are representative for n≥ 8 independent animals per strain (mean ± SEM; *p<0.05; **p<0.01; ***p<0.001).

Respiratory DC represent a heterogenous population of professional antigen presenting cells. Accordingly we performed 7-color flow cytometry to quantitate the four major DC subsets in different inbred mouse strains (Figure 3, Figure 4). Multicolour flow cytometry allowed us to separate four respiratory DC subpoulations, plasmacytoid DC (pDC; 120g8+, CD11c+, Siglec F^neg^, CD49b^neg^, CD45+), CD103+ DC (CD103+, 120g8^neg^; CD11c+; Siglec F^neg^, CD49b^neg^, CD45+), monocytic DC (moDC; CD11b^high^, MHC-II^med-low^, CD11c+; Siglec F^neg^, CD49b^neg^, CD103^neg^, 120g8^neg^ CD45+), and CD11b^high^ DC (CD11b^high^, MHC-II^high^, CD11c+; Siglec F^neg^, CD49b^neg^, CD103^neg^, 120g8^neg^ CD45+). 129SV mice exhibited significantly elevated pDC and CD11b^high^ DC numbers (p<0.001 and p=0.0003; Figure 4A, D) when compared to C57BL/6, C3H, DBA or BALB/c mice. In contrast CD103+ DC and MoDC numbers were significantly elevated in C3H mice when compared to C57BL/6, BALB/c or DBA strains (p<0.01; Figure 4B, C). These results suggested significant strain heterogeneity of functional divergent DC subsets. 129SV mice can be characterized as rich in pDC and CD11b^hi^ DC. In contrast, C3H mice are rich in CD103^+^ DC and MoDC. Magnitude of DC subset fold-differences for all strains in comparison to C57BL/6 mice is summarized in Table 2.

### Inbred mouse strains show marked differences of respiratory T and B cells, innate lymphocytes and Lin^neg^ leukocytes

**Figure 5:**
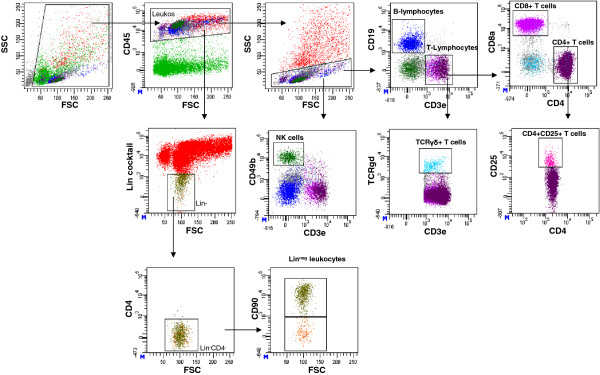
**Gating strategy for identification of respiratory lymphocyte subsets and Lin**^**neg**^**leukocytes.** Respiratory B and T lymphocytes were identified according to low side scatter characteristics, CD19 and CD3e surface expression. This gating strategy resulted also in exclusion of autofluorescent respiratory macrophages. Out of the CD3e-positive population CD4+ T helper, CD4^+^CD25^+^ T helper and CD8 T cells were gated according to staining with the respective mAbs and γδ T cells were identified by using a γδ TCR mAb. Please note that the CD4^+^CD25^+^ T cells are not CD25^high^. A more detailed gating strategy of Treg subsets including isotpye controls is shown in Figure 8 . Natural killer cells were identified according to CD49b^+^Cd3e^neg^ expression. Respiratory lineage-negative leukocytes were identified by absence of CD3e, CD8a, CD11b, CD11c, CD19, B220, TER-119, FceR1 (Lineage cocktail) and CD4 expression and subsequently separated into CD90^+^ and CD90^neg^ cells.

**Figure 6:**
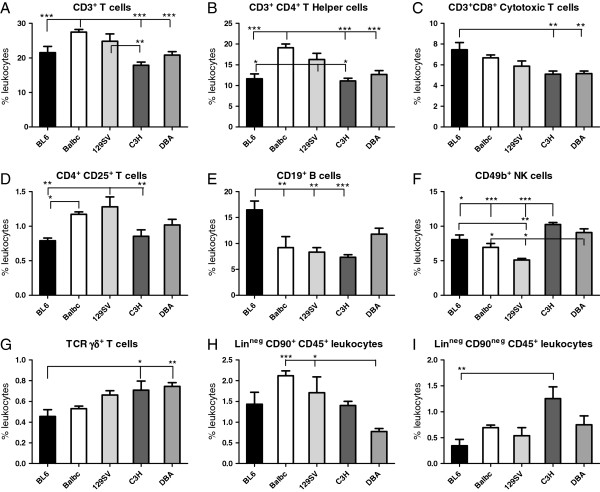
**Significant strain heterogeneity of respiratory T and B lymphocytes, NK cells, γδ T cells and Lin**^**neg**^**leukocytes.** Dissection of respiratory lymphocyte subsets suggests elevated CD3 T cell and CD4 T helper cell numbers in BALB/c mice (**A**, **B**). CD8+ T cell frequencies are elevated in C57BL/6 (BL6) mice when compared to either C3H or DBA mice (**C**). Putative T regulatory cell (CD4+CD25+ T cells) numbers are significantly elevated in 129SV mice compared to BL6 or C3H strains (**D**). Please note that the CD4^+^CD25^+^ T cells are not CD25^high^. A more detailed gating strategy of Treg subsets including isotpye controls is shown in Figure 8. B lymphocyte numbers are significantly elevated in BL6 mice (**E**). Natural killer (NK) cells and γδ T cell numbers are elevated in C3H and DBA strains (**F**, **G**). BALB/c and 129SV mice are rich in Lin-^neg^ CD90^+^ leukocytes (**H**) whereas C3H mice exhibit high numbers of Lin-^neg^ CD90^neg^ leukocytes (**I**). Results are representative for n≥ 8 (**A**, **B**, **C**, **F**, **G**) or n ≥ 5 (**D**, **E**) independent animals per strain (mean ± SEM; *p<0.05; **p<0.01; ***p<0.001).

Classical B and T lymphocyte subsets were identified after multiparameter gating for standard surface markers (Figure 5). The two major innate lymphocyte subsets NK cells and γδ TCR+ lymphocytes were identified according to CD49b (CD3^neg^ CD49b^+^) and γδ TCR expression (CD3^pos^ γδ TCR ^+^), respectively (Figure 5). The NK1.1 mAb was not selected to identifiy NK cells, because the antigen is not expressed on BALB/c, DBA, C3H and 129SV mice. Classical CD3+ T cells as well as CD4+ T helper cell numbers were significantly elevated in BALB/c mice when compared to C57BL/6, C3H or DBA mice, whereas CD8+ T cells showed no major strain differences (Figure 6A-C). CD4+CD25+ putative Treg cell numbers were significantly reduced in C57BL/6 mice when compared to either BALB/c or 129SV mice (Figure 6D). Since CD4+CD25+ T cells are not proven Treg cells, we performed additional analyses of Foxp3+ Treg subsets (please see detailed data at the end of results).

With respect to respiratory B cells, C57BL/6 mice exhibited significant elevation of respiratory CD19+ lymphocyte numbers when compared to any other inbred mouse strain (Figure 6E). Flow cytometry analysis revealed significantly elevated NK cell numbers in C3H mice versus C57BL/6, BALB/c and 129 SV mice (Figure 6F). With respect to respiratory γδ T cells, C3H mice also exhibited the highest numbers, but strain heterogeneity was less pronounced (Figure 6G). Lin^neg^ respiratory leukocytes were stratified according to CD90 expression and showed also strain heterogeneity (Figure 6 H,I). The highest Lin^neg^ CD90^+^ leukocyte numbers were detectable in BALB/c mice (2.1% ± 0.1) and the lowest numbers in DBA mice (0.7% ± 0.07; p<0.001). In contrast, Lin^neg^ CD90^neg^ leukocyte numbers elevated in C3H mice (1.2% ± 0.22) and decreased in C57BL/6 mice (0.3% ± 0.12; p<0.001). These results indicated marked strain heterogeneity of respiratory lymphocytes and innate leukocyte numbers with major differences between C57BL/6, BALB/c and 129 SV mice. Lungs of C57BL/6 mice can be characterized as B cell “rich”, whereas BALB/c and 129SV mice are rich in T cells. With respect to innate lymphocytes, C3H and DBA mice can be characterized as rich in NK cells and γδ T cells. Magnitude of lymphocyte subset fold-differences in comparison to C57BL/6 mice is summarized in Table 2.

### Cytokine production after stimulation of respiratory leukocytes from inbred mouse strains

**Figure 7:**
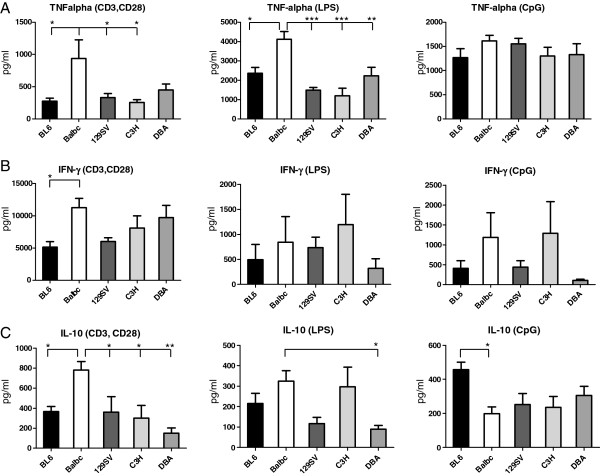
**Strain heterogeneity of respiratory TNF-α, IFN-γ and IL-10 production.** Respiratory leukocytes from inbred mouse strains were purified with CD45-microbeads. TNF-α (**A**), IFN-γ (**B**) and IL-10 (**C**) production were analyzed after T cell (CD3/CD28 mAb), TLR4 (LPS) and TLR9 (CpG ODN 1826) stimulation. BALB/c mice produced significantly higher amounts of TNF-α both after T cell and TLR4 stimulation (**A**) and higher IFN-γ production after T-cell stimulation when compared to C57BL/6 (BL6) mice (**B**). IL-10 production was significantly elevated in BALB/c mice after T cell stimulation and LPS stimulation whereas C57BL/6 (BL6) mice produced significantly more IL-10 after TLR9 stimulation when compared to BALB/c mice (**C**). Results are representative for n≥ 5 independent animals per strain (mean ± SEM; *p<0.05; **p<0.01; ***p<0.001).

Given the different composition of respiratory leukocyte subsets we questioned whether TNF-α (Figure 7A), IFN-γ (Figure 7B) and IL-10 (Figure 7C) production of respiratory leukocytes also exhibited strain heterogeneity. To address different pathways and cell types we stimulated respiratory T lymphocytes via CD3/CD28 mAbs and TLR4 and TLR9 expressing leukocytes via their specific ligands, LPS and CpG ODN. Results indicated that BALB/c mice produced significantly higher amounts of TNF-α both after T cell stimulation and after LPS stimulation than any other strain (Figure 7A). In contrast, after TLR9 stimulation TNF-α production showed no significant strain heterogeneity. IFN-γ production was significantly elevated in BALB/c mice versus C57BL/6 mice after T cell stimulation (Figure 7B). With respect to the anti-inflammatory cytokine IL-10, BALB/c mice produced significantly more IL-10 after T cell stimulation than any other strain (Figure 7C). In contrast, after TLR9 stimulation C57BL/6 mice produced significantly more IL-10 in comparison to BALB/c mice. These results indicated that strain heterogeneity exists also with respect to cytokine production capacity. Respiratory leukocytes from BALB/c mice showed a trend towards high cytokine production whereas DBA mice in most of the experiments produced low cytokine levels.

### Significant differences of respiratory Foxp3^+^ Treg cells across inbred mouse strains

**Figure 8:**
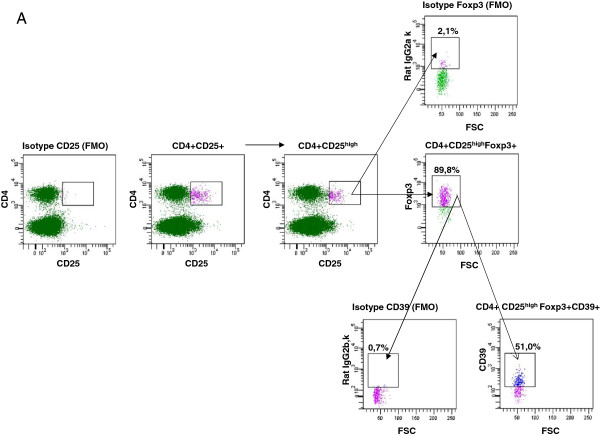
**Gating strategy for identification of respiratory Treg subsets.** CD4-positive cells were gated over CD25 (**A**). Depending on CD25-expression, CD4^+^ CD25^+^ and CD4^+^ CD25^high^ populations were detected. Out of the CD4^+^ CD25^high^ fraction, Treg cells were identified based on Foxp3 expression. Out of the CD4^+^ CD25^high^ Foxp3^+^ regulatory T cell fraction, a CD39^+^ subset was identified (**A**). CD4^+^ CD25^+^ CD127^low/neg^ Tregs were identified after gating of CD4^+^ cells over CD127 (**B**). Subsequently, the CD4^+^ CD127^low/neg^ fraction was gated over CD25. To confirm, that most of the CD4^+^ CD25^+^ CD127^low/neg^ cells were Tregs, they were finally gated over Foxp3 (**B**). Isotype mAb controls were used in all gates to control for unspecific background staining. Fluorescence minus one (FMO) isotype controls were used to set the gates for CD25, Foxp3, CD39 and CD127 positive subpopulations.

**Figure 9:**
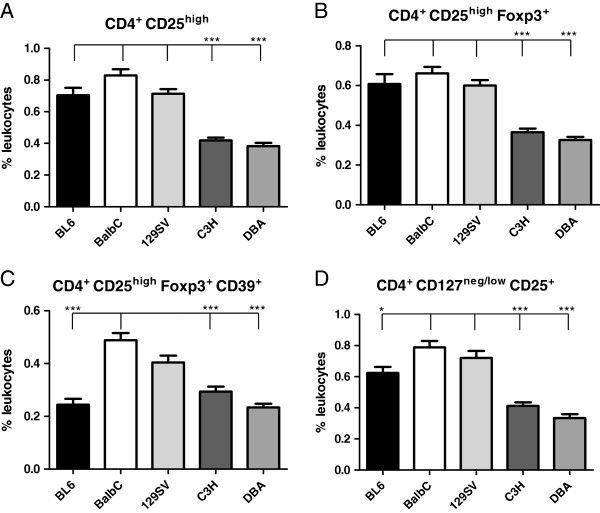
**Inbred mouse strains exhibit different numbers of respiratory Treg cells.** Analysis of Treg populations according to CD25, Foxp3, CD39 and CD127 expression indicates significantly increased CD4^+^ CD25^high^ (**A**), CD4^+^ CD25^high^ Foxp3^+^ (**B**), CD4^+^ CD25^high^ Foxp3^+^ CD39^+^ (**C**), and CD4^+^ CD25^+^ CD127^low/neg^ (**D**) Treg subsets in BALB/c mice in comparison to C3H and DBA mice. Results are representative for n≥ 5 independent animals per strain (mean ± SEM; *p<0.05; ***p<0.001).

Given the increased numbers CD4^+^ CD25^+^ in BALB/c and 129 SV mice we questioned whether these cells represented Treg cells and extended our analysis. Based on CD25, Foxp3, CD39 and CD127 expression we analysed the following four Treg subsets: CD4^+^ CD25^high^, CD4^+^ CD25^high^ Foxp3^+^, CD4^+^ CD25^high^ Foxp3^+^ CD39^+^ (Figure 8A) and CD4^+^ CD25^+^ CD127^low/neg^ (Figure 8B) Treg populations. Foxp3^+^ expression of the CD4^+^ CD25^+^ CD127^low/neg^ Tregs was controlled by intracellular staining and found to be >90% positive (Figure 8B). Quantitative results indicated that BALB/c and 129 SV strains exhibited significantly higher numbers of CD4^+^ CD25^high^, CD4^+^ CD25^high^ Foxp3^+^ and CD4^+^ CD25^+^ CD127^low/neg^ Tregs in comparison to C3H and DBA strains (Figure 9 A-D). C57BL/6 mice exhibited similar Treg numbers in comparison to BALB/c mice with the exception of the CD4^+^ CD25^high^ Foxp3^+^ CD39^+^ and CD4^+^ CD25^+^ CD127^low/neg^ populations (Figure 9 C, D). Magnitude of Treg subset fold-differences in comparison to C57BL/6 mice is summarized in Table 2.

## Discussion

In this report we demonstrate marked strain heterogeneity of respiratory leukocyte subsets in five major inbred mouse strains. To our knowledge this is the first analysis that has quantitated DC subsets, macrophages, classical lymphocyte subsets, Treg subsets, innate lymphocytes including γδ T cells and lin^neg^ leukocytes in the respiratory tract. Environmental effects are unlikely to account for these differences, since specific-pathogen free age-matched animals were received from the same provider and housed under specific-pathogen free conditions. The enumerated leukocyte subsets are essential for different immunological tasks and therefore it is possible that the observed strain heterogeneity influences respiratory immunity to different pathogens.

With respect to respiratory DC subsets we found a striking elevation of respiratory pDC in 129SV mice in comparison to all other strains. These results extend the finding of Asselin-Paturel et al., and Nakano et al. who reported significantly elevated pDC frequencies in spleen and blood of 129SV mice [20,21]. Since respiratory pDC represent a key DC subset responding to viral infection and additionally play an immunoregulatory role by preventing airway hyperresponsiveness, higher pDC numbers may influence pathogenesis of respiratory allergy and viral infection in 129SV mice versus other strains. With respect to respiratory CD103^+^ DC our results indicated that C3H mice exhibited significantly higher numbers in comparison to 129Sv and DBA mice. Given the critical role of respiratory CD103^+^ DC for activation of naive CD8+ T killer cells in respiratory viral infections [5,22], different CD103+ DC numbers may impact the strain-dependent capacity to generate viral CD8^+^ T cell immunity. Moreover, differences have also been reported according to DC endocytosis receptor expression, such as mannose receptor [23] and CD207 (langerin, a c-type specific lectin) [24] highlighting separate levels of strain heterogeneity. In line with these reports, our analysis of respiratory leukocytes indicated significant strain differences to produce TNF-α and IL-10 that is partially dependent on the selected Toll-like receptor ligand and the activated leukocyte subset. Our results revealed that respiratory leukocytes of BALB/c mice produced the highest TNF-α levels both after TLR4 stimulation through LPS and after T-cell stimulation through CD3/CD28 mAbs. These results support the finding of Gosselin et al. demonstrating that resistance of BALB/c mice to pseudomonas aeruginosa is dependent on increased TNF-α production [25].

In addition to significant differences of DC subsets and cytokine production capacity we found marked differences of respiratory Foxp3^+^ Treg frequencies across different inbred strains. In general terms, BALB/c and 129SV mice exhibited significantly higher respiratory Treg numbers than C3H and DBA mice. Respiratory Treg elevation was evident with respect to four different Treg subsets, namely CD4^+^ CD25^high^, CD4^+^ CD25^high^ Foxp3^+^, CD4^+^ CD25^high^ Foxp3^+^ CD39 and CD4^+^ CD25^+^ CD127^low/neg^ Tregs. Given the important role of Treg in immunoregulation, additional studies are required to dissect the genetic cause und functional relevance of Treg differences in inbred mouse strains.

Both numerical and functional leukocyte strain variabilities are likely to contribute to innate resistance and susceptibility to infection with various pathogens. Examples are the unique susceptibility of DBA/2 mice to pulmonary tuberculosis as well as resistance of 129S6/SVevTac mice to salmonella typhimurium infection [26]. Genetic dissection of strain heterogeneity promoted the discovery of several highly relevant immune response genes and modifiers underlining the importance of inbred mouse models to understand innate and adaptive immunity [26,27].

Furthermore, knowledge of significant numerical differences of respiratory leukocyte subsets is also practically relevant for the planning and setup of ex vivo experiments. Several functionally distinct cells, like pDC, represent extremely rare subsets in the lung and therefore ex vivo analysis of sufficient numbers of purified respiratory pDC is technically difficult. Accordingly, our results may help identification of the suitable inbred mouse strain with the highest pDC numbers. Given pDC frequencies in the five major inbred strains ranging from 0.15% (mean of C3H mice) to 0.58% (mean of 129SV mice) of leukocytes while having similar total leukocyte counts, selection of the 129SV mouse would increase the pDC yield 3 to 4-fold and would save both animal numbers and procedural time. Similar conclusions can be drawn with respect to sorting of rare Lin^neg^ leukocytes populations from the lungs for ex vivo experiments. Our results indicated that C57BL/6 mice have only few Lin^neg^ CD90^neg^ leukocytes (mean 0.34% of leukocytes) in comparison to C3H mice (mean 1.25 %).

In more general terms, our study indicated characteristic respiratory immunophenotypes of inbred mouse strain: BALB/c mice are rich of eosinophils and Treg cells; C57BL/6 mice are rich of B cells and low in eosinophils. In contrast, 129SV mice are high in pDC and Tregs and C3H mice are high in CD103^+^ DC, NK cells and γδ T cells. These characteristic immunophenotypes may help the selection of strains for respiratory disease models, e.g. if the analysis and purification of respiratory pDC during viral pneumonia is required then 129SV mice would be a suitable model. However, besides different respiratory immunophenotypes, many other functionally relevant strain differences have been reported. Therefore, careful strain selection based on disease model and published strain differences may help to select the best mouse strain representing human phenotypes. In this context, DeVooght et al. recently reported, that choice of mouse strain significantly influenced the outcome in a model of chemical-induced asthma. They reported that the human phenotype of chemical-induced asthma was best reproduced in BALB/c when compared to 6 other strains [28].

Inbred mouse strain differences have been reported with respect to different aspects of inflammation. With respect to the respiratory irritant ozone, C57BL/6 mice have been reported relatively susceptible when compared to relatively resistant C3H mice [29]. With respect to chronic pseudomonas aeruginosa infection, BALB/c mice are resistant in comparison to highly susceptible DBA/2 mice [30]. Similarly, in a pneumovirus model, BALB/c and C57BL/6 strains were reported to be relatively resistant in comparison to DBA/2 or 129SV mice [31].

In summary, the present study highlights marked numerical differences of several important leukocytes subsets in the lungs of major inbred mouse strains. Although our results are primarily descriptive, it may provide the basis for additional functional studies under inflammatory in vivo conditions. Variation of respiratory immune responses and cytokine production capacity may partially be related to strain differences in respiratory leukocyte composition and function.

## Competing interests

The authors declare that they have no competing interests.

## Authors’ contributions

HH designed research, analyzed and interpreted data and wrote the manuscript; AW, NB, SK performed research, analyzed and interpreted data; JL and GB interpreted data and critically read the manuscript. All authors have read and approved the final manuscript.
